# Exercise-Induced Modulation of Baroreflex Control of Sympathetic Nerve Activity

**DOI:** 10.3389/fnins.2018.00493

**Published:** 2018-07-23

**Authors:** Kenju Miki, Misa Yoshimoto

**Affiliations:** Department of Environmental Health, Life Science and Human Technology, Nara Women’s University, Nara, Japan

**Keywords:** exercise, baroreflex, sympathetic nerve activity, post-exercise hypotension, orthostatic intolerance

## Abstract

Exercise modulates arterial pressure (AP) regulation over various time spans. AP increases at the onset of exercise and this increase is then sustained during exercise. Once exercise is stopped, AP is suppressed for up to an hour afterwards. Prolonged endurance training is associated with dysfunction of the sympathetic regulation of AP in response to posture changes (orthostatic intolerance). Baroreflex control of sympathetic nerve activity (SNA) has been extensively studied to understand the mechanisms underlying exercise-induced changes in AP. We have previously presented entire baroreflex AP-SNA curves during and after exercise, and during central volume expansion, obtained using direct measurements of renal sympathetic nerve activity (RSNA) in conscious animals. In this review, we describe the modulatory effects of exercise on baroreflex control of AP based on these entire AP-RSNA baroreflex curves. We suggest that both acute and chronic exercise can have modulatory effects on the entire baroreflex curve for SNA, and that these effects differ among time periods.

## Introduction

Exercise modulates the autonomic regulation of arterial pressure (AP). Immediate and simultaneous increases in AP, sympathetic nerve activity (SNA), and heart rate (HR) occur during exercise (Miki et al., 2003; Fadel, 2015) and both AP and SNA transiently decrease after cessation of acute bouts of exercise in animals and humans (Halliwill et al., 1996a; Miki et al., 2003). In addition, chronic endurance exercise is frequently associated with adverse effects on AP regulation in response to postural changes (Greenleaf et al., 1981; Winker et al., 2005). The cardiovascular responses to exercise are mediated via multiple, complexly integrated, neuronal and humoral inputs that coordinate cardiovascular function. For more detail, readers are referred to excellent reviews from different perspectives, including central mechanisms (Potts, 2001; Raven et al., 2006; Matsukawa, 2012; Fisher et al., 2015; Dampney, 2017). Among the multiple regulatory systems involved in coordinating cardiovascular functions, the arterial baroreflex is thought to play a primary role in modulating and coordinating the cardiovascular response to exercise (Miki and Yoshimoto, 2013; Dampney, 2017). Although the potential contribution of the arterial baroreflex to AP regulation during and after acute and chronic exercise has been studied extensively, the mechanism by which exercise modulates baroreflex control of SNA remains unclear.

Studies of the effects of acute and chronic exercise on baroreflex control of SNA have yielded inconsistent results. These inconsistencies may have several causes. First, measurement of SNA during exercise has methodological limitations. Comparisons of basal SNA levels among different groups or preparations inevitably include non-physiological variation (Hart et al., 2017). Because SNA is measured by contacting an electrode to a nerve bundle that contains many axons the signals obtained comprise multiple spikes. The basal SNA signal is affected by factors such as the number of axons in the bundle and the electronic resistance between the axon and electrode; these vary among individual subjects and surgical preparations in both human and animal experiments. In our experience in animal studies, there is huge individual variation in the raw units of voltage, burst height, and frequency of renal SNA (RSNA) and lumbar SNA (LSNA) measurements. This variation occurred even though the electrode implantation surgery was performed by a single experienced researcher and the experimental environment, including the amplifier used for SNA recording, the electrical shielding, and the ambient temperature, was identical for all animals. Thus, comparing baselines among different subjects or animals may lead to inconsistent results because of unquantifiable differences in the relationship between the electrodes used for measurement and the neurons being measured. Longitudinal studies, in which data are collected from the same animal/subject or experimental preparation, may be more suitable than cross-sectional studies for comparing relative changes in SNA induced by exercise.

Second, data on baroreflex gain for SNA has been fitted to either a simple linear equation or a logistic sigmoid function (Kent et al., 1972). The logistic sigmoid function can describe the full range of the stimulus-response curve for the arterial baroreflex. It is written as:

Y=A1/{1+exp[A2(X−A3)]}+A4,

where *Y* is RSNA or HR, *X* is AP, A1 is the response range for *Y* (upper plateau minus lower plateau), A2 is the gain coefficient, and A3 is the pressure at the midrange of the curve (centering point). The centering point is the point at which there is an equal depressor and pressor response to a given change in AP. A4 is the lower plateau of *Y*.

In human experiments, because it is difficult to fluctuate AP over its full range (Halliwill et al., 1996a), a linear equation is commonly used to assess baroreflex function. The primary disadvantage of the linear equation method is that the range of changes in AP used to estimate baroreflex function in this equation do not cover the entire range of potential AP fluctuations. This method underestimates the gain coefficient for AP changes near either the lower or upper plateau (Dampney, 2017). To assess the full extent of exercise-induced modulation of baroreflex control of AP the entire baroreflex curve should be generated.

In this brief review we describe modulation of the entire baroreflex stimulus-response curve for RSNA during exercise and after cessation of exercise. In addition, we discuss the possible role of central blood volume expansion, which is commonly seen in endurance athletes, in inducing changes in the entire RSNA baroreflex curve. The data discussed in this review were generated in our laboratory by continuous and simultaneous measurement of AP, RSNA, and HR. All variables were measured in all experimental rats, and the measurement electrodes were placed in all animals by the same experienced researcher (Miki et al., 2003; Nagura et al., 2004). These longitudinal studies have given us a quantitative insight into exercise-induced modulation of baroreflex control of SNA.

## During Exercise: Right and Upward Shifts of the Baroreflex Curve

**FIGURE 1:**
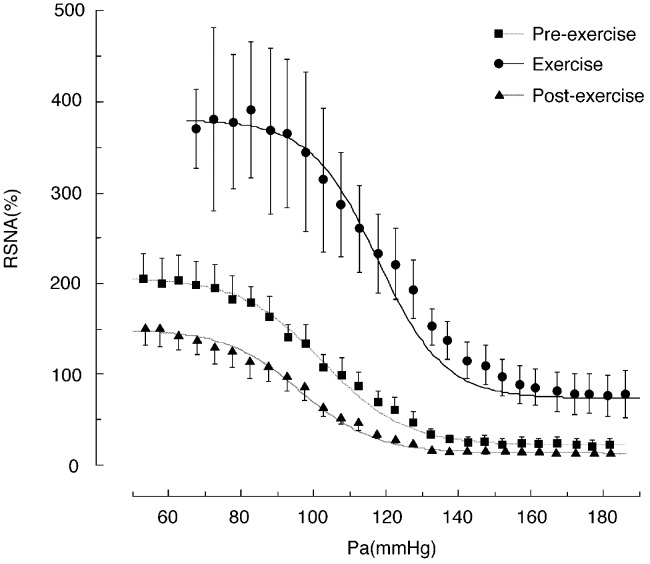
Baroreflex curves for renal sympathetic nerve activity (RSNA) obtained during the pre-exercise, treadmill exercise, and post-exercise periods. Pa, arterial pressure. Symbols and bars indicate means ± SEM, respectively. From Miki et al. (2003) with permission.

The onset of exercise causes immediate and simultaneous increases in AP, SNA, and HR (Rowell, 1986; Fadel, 2015). It has been consistently reported that the magnitude of the increases in SNA and HR are proportional to the intensity of the exercise (Miki et al., 2003; Boulton et al., 2014). The magnitude of the increase in AP is also likely proportional to the shift in the baroreflex curve for SNA. We generated the entire baroreflex curve for RSNA during treadmill exercise and during grooming (a mild and voluntary exercise in rats) (Miki et al., 2003; Nagura et al., 2004; Boulton et al., 2014) and found that exercise shifts the entire baroreflex curve for RSNA to the right and upward (**Figure 1**).

The upward shift in the baroreflex curve for RSNA observed during treadmill exercise was characterized by an approximately twofold increase in the upper plateau (from 210 to 380% of the pre-exercise level, shown in **Figure 1**) and an approximately 50% increase in the lower plateau (Miki et al., 2003). Grooming, which is a relatively mild exercise in comparison to treadmill running, caused an approximately 50% increase in the upper plateau without significant changes in the lower plateau (Nagura et al., 2004). This suggests that the magnitude of the upward shift may depend on exercise intensity. The rightward shift of the baroreflex curve for RSNA observed during treadmill exercise in rats was characterized by significant increases of approximately 20 mmHg in both the AP saturation and threshold pressures, without significant changes in operating pressure (Miki et al., 2003). Similarly, grooming caused increases of approximately 10 mmHg in the AP saturation and threshold pressures without significant changes in operating pressure (Nagura et al., 2004). Thus, the magnitude of the rightward shift in the baroreflex curve for RSNA may also depend on exercise intensity. These results suggest that exercise intensity may play a critical role in determining the magnitude of the upward and rightward shifts of the baroreflex curve for RSNA and these exercise-induced increases in RSNA and AP are in proportion to the exercise intensity.

The exercise-induced shift in baroreflex control of SNA may allow increased AP to be stably maintained despite the massive and variable vasodilation that occurs with dynamic exercise (Miki et al., 2003; Fadel, 2015; Drew, 2017). This may be achieved through the increase in baroreflex gain without a concomitant decrease in operating range, and by the relocation of operating pressure to around the midpoint pressure of baroreflex curve (Miki et al., 2003). It should be noted that the AP operating range moves toward high pressure during exercise. SNA and HR can respond to cause compensatory increases or decreases only when AP changes within the operating range. When AP is outside of the operating range, both SNA and HR become constant and do not respond to changes in AP, thus losing regulatory capacity. Because AP changes so rapidly during exercise, maintaining an appropriately wide operating range is critical to allow normal physiological regulatory mechanisms to function. Maintenance of the operating range is accompanied by relocation of the operating pressure to near the centering point where gain is maximal; at this point the system can respond equally to an increase or decrease in AP. These changes in gain and operating range, and relocation of the operating pressure, allow high AP to be maintained and stabilize any exercise-induced pressure fluctuations.

The changes in baroreflex gain in response to exercise have been discussed extensively. In simple linear regression analysis of the baroreflex function, the slope of the regression line has been used as an index of baroreflex gain. However, gain is just one of the parameters that describe the baroreflex curve. An increase in gain does not always imply improved AP regulation. The entire baroreflex curve should be generated to fully understand the ability of the baroreflex to buffer against exercise-induced changes in AP.

## The Exercise-Induced Shift in the Baroreflex Curve for Hr Is Different From That for Rsna

**FIGURE 2:**
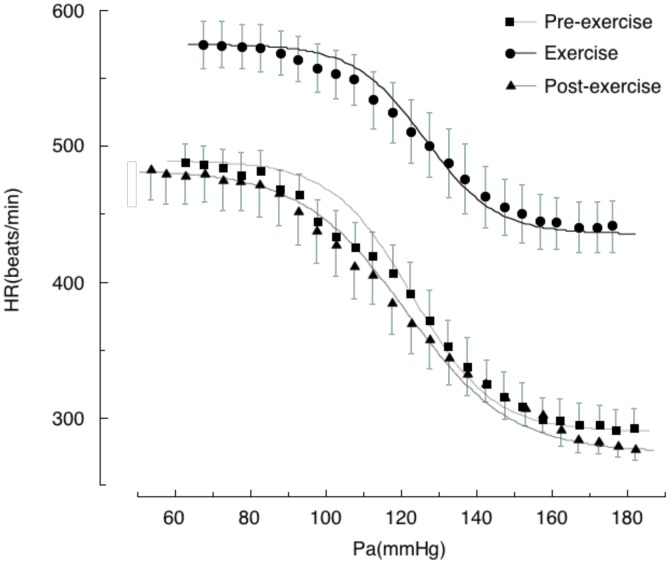
Baroreflex curves for heart rate (HR) obtained during pre-exercise, treadmill exercise, and post-exercise periods. Symbols and bars indicate means ± SEM, respectively. Pa, arterial pressure. From Miki et al. (2003) with permission.

We generated baroreflex curves for HR and RSNA simultaneously in the same rats during and after treadmill exercise and during REM sleep (**Figures 1**, **2**) (Miki et al., 2003). The baroreflex curves for HR differed from those for RSNA in all three time periods. Exercise caused a vertical shift in the baroreflex curve for HR without a rightward shift; this is consistent with previous reports (Potts et al., 1993). Furthermore, the AP–HR baroreflex curve obtained during the post-exercise period was identical to that obtained during the pre-exercise period while the AP–RSNA baroreflex curve was suppressed vertically during the post-exercise period (**Figures 1**, **2**). It is clear that the changes in baroreflex control of HR are not representative of the changes in whole body baroreflex function during exercise. Thus, the baroreflex curve for HR represents a distinct baroreflex pathway that only controls HR. Arterial baroreflex control is a multi-input, multi-output, and multilevel control system (Sagawa, 2011) and it is necessary to specify the dependent variable for the baroreflex curve when shifts in baroreflex curves are discussed. Simple extrapolation of results from a baroreflex curve for HR to variables such as SNA could lead to inconsistent conclusions.

## Post-Exercise Hypotension: Vertical Suppression of the Baroreflex Curve for Rsna

Cessation of exercise causes a transient reduction in AP that is referred to as post-exercise hypotension (PEH) (Kenney and Seals, 1993; Chen and Bonham, 2010; Romero et al., 2017). This has been observed following exercises such as walking, running, cycling, swimming, and resistance training (Chen and Bonham, 2010). The magnitude of the PEH depends on resting AP and the intensity and duration of the exercise (Halliwill, 2001). PEH is due to a persistent decrease in systemic vascular resistance that is not completely offset by increased cardiac output, and has a neural component that is thought to be a reduction in SNA. Reductions in SNA of approximately 30% from basal levels in humans (Halliwill, 2001) and reductions in RSNA of approximately 25% in rats (Miki et al., 2003) have been reported after exercise.

The SNA suppression that occurs during PEH suggests that baroreflex control of SNA is modulated after exercise (Halliwill et al., 1996a; Kajekar et al., 2002; Miki et al., 2003; Willie et al., 2011). Using rats, we generated entire AP-RSNA baroreflex curves before and after a single session of treadmill exercise at approximately 70% VOmax (**Figure 1**) (Miki et al., 2003). The AP-RSNA baroreflex curve was suppressed vertically after exercise; the upper plateau was reduced by approximately 50% without any alterations in the minimum response or the gain coefficient. This suggests that RSNA after exercise is lower at all levels of AP than during the pre-exercise period, which results in a lower AP during daily activities after exercise. Consistent with this view, in humans a decrease in AP has been shown to persist while performing normal activities of daily living after exercise (MacDonald et al., 2001).

The reduction in the maximum response of the AP-RSNA curve that occurs following exercise may be centrally controlled. Kajekar et al. (2002) reported that the maximum unit activity of the rostral ventrolateral medulla (RVLM) was significantly attenuated during PEH. They further demonstrated that this reduction in RVLM neuronal activity was mediated, at least in part, by GABA_A_–receptor signaling (Kajekar et al., 2002; Chen and Bonham, 2010). It is likely that post-exercise inhibition of sympathetic motoneurons in the RVLM via GABA receptors attenuates the maximum RSNA during the post-exercise period.

Although the mechanisms underlying tonic suppression of RVLM sympathetic neurons after exercise is not clear, the following evidence offers some insight into its origins. PEH has been observed after various types of large-muscle dynamic exercise including walking, running, cycling and swimming, and after exercise to exhaustion. Electrical stimulation of the sciatic nerve, as well as the gastrocnemius and biceps femoris muscles, elicits post-stimulation hypotension in anesthetized animals (Kenney et al., 1991). These data suggest that large muscle contractions, and possibly associated somatic afferent nerve activation, may trigger PEH. Higher CNS activity (central command) likely plays only a minor role in PEH because the hypotensive response to exercise has been observed in animals under anesthesia. In addition, the AP nadir during PEH occurs within the first hour of recovery from exercise (Kenney and Seals, 1993). This suggests that clearance of the neurotransmitters that cause the greatest inhibition of RVLM sympathetic pre-motoneurons may occur within an hour after cessation of exercise. It is likely that the increase in somatic afferent activity caused by muscle contraction modulates inhibition of the RVLM for more than an hour, causing suppression of the upper plateau of RVLM activity such that the AP-RSNA curve is shifted downward. In this way, RSNA is suppressed for more than an hour after exercise, inducing PEH.

## Orthostatic Intolerance: Volume Expansion Acts as an On-Off Modulator of the Baroreflex Curve for Rsna

Greenleaf et al. (1981) stated that “trained men can run, but they cannot stand”. Endurance-trained athletes have a diminished tolerance to orthostatic stress (Greenleaf et al., 1981; Levine et al., 1991; Winker et al., 2005; Lucas et al., 2008). Orthostatic intolerance (OI) can be defined as the inability to tolerate an upright posture, relieved by recumbency. When the posture changes from supine to upright, central blood shifts to the lower body due to gravity; this causes decreases in venous return, end-diastolic volume, and cardiac output, and results in orthostatic hypotension (Rowell, 1986). When AP decreases after a postural change the baroreflex triggers immediate increases in SNA; these increase total peripheral resistance, heart rate, and cardiac contractility and maintain AP within its appropriate range (Stewart, 2012; Iwase et al., 2014). The initial AP drop after postural change, denoted “initial orthostatic hypotension,” is finished within 30–60 s and appropriate blood pressure is rapidly restored (Stewart, 2012). OI is recognized as autonomic failure attributable to an inadequate sympathetic vasoconstrictive response (Stewart, 2012, 2013). The active compensatory vasoconstrictive response, which is normally triggered by the baroreflex within seconds of a postural change, does not work appropriately in OI. This results in an AP reduction and can lead to syncope in endurance-trained athletes. The underlying cause of the failure of this compensatory mechanism is unknown.

Chronic endurance training is associated with an increase in plasma volume (Convertino, 1991), which should reduce OI by attenuating the reduction in venous return that occurs after transition to an upright posture. Improved venous return should improve cardiac output and prevent an abrupt drop in AP. However, this has not proven to be the case; instead, the increase in plasma volume induced by chronic endurance training has been associated with the occurrence of OI. It has been suggested that symptoms of OI in highly fit subjects might be caused by attenuated carotid baroreflex responses (van Lieshout, 2003), but how the baroreflex curve for SNA is modulated in endurance athletes remains unclear.

**FIGURE 3:**
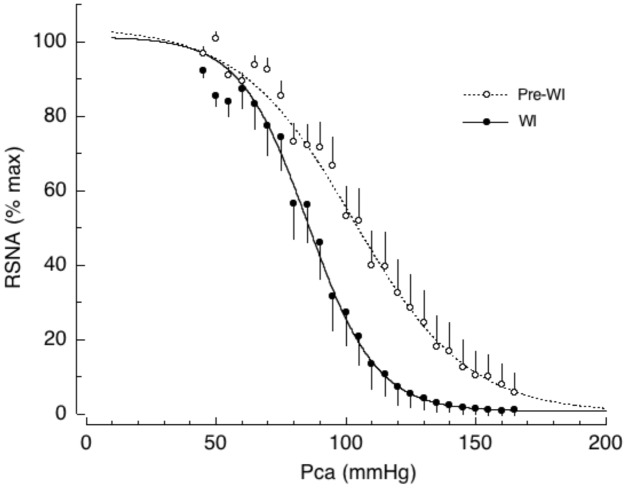
Baroreflex curves for renal sympathetic nerve activity (RSNA) during cardiopulmonary baroreceptor loading induced by head out water immersion (WI, solid lines). WI has been used as an investigative tool to cause acute loading of cardiopulmonary baroreceptors without administration of either drugs or fluid. Pre-WI, pre-water immersion (dotted lines); Pca, carotid arterial pressure. Symbols and bars indicate means ± SEM, respectively. From Miki et al. (2009) with permission.

To explain the initial inadequate vasoconstriction in response to posture change, we propose a hypothesis based on observations in conscious dogs during water immersion (WI). This hypothesis explains, in part, why plasma volume expansion does not protect against, and may in fact trigger, OI (Miki et al., 2009). Head-out WI causes increased central blood volume because of relocation of blood from the extremities to the cardiopulmonary area due to the hydrostatic pressure of the water. WI also increases plasma volume due to transcapillary fluid shifts from the interstitial to the intracapillary space. Thus, WI allows us to generate the entire baroreflex curve in response to blood volume expansion in the same animal without administration of external fluid. We found that the central blood volume expansion caused by WI shifts the AP-RSNA baroreflex curve to the left causing a significant decrease in the saturation pressure of the AP response and reducing the operating range by half, while increasing the gain coefficient and maximal gain (**Figure 3**, Miki et al., 2009). In addition, WI reset the operating pressure to near the saturation pressure of AP. Although the baroreflex gain increased, overall this shift would be disadvantageous for maintaining a stable pressure in the face of rapid decreases in AP for several reasons. First, the operating range decreased by half, which would fail to drive linear compensatory feedback mechanisms in the face of abrupt AP changes. Second, the operating pressure was reset to near the saturation pressure of AP, where baroreflex gain is low. It is, therefore, likely that central volume expansion acts as a “Flip-Flop” or “On-Off”-type modulator of the baroreflex AP-RSNA curve. Compared with linear control systems, Flip-Flop- or On-Off-type regulatory systems (such as bimetallic thermostats) provide rather unstable output, which is characterized by responses that overshoot and undershoot the optimal (desired) level. The low baroreflex gain causes AP to fluctuate in response to external disturbances. The baroreflex-resembling Flip-Flop-type regulator is therefore not likely to immediately excite SNA, thus causing a delay in compensatory cardiovascular responses against the abrupt decrease in AP. Thus, the chronic increase in central blood volume that occurs with endurance training can explain, at least in part, training-associated OI.

## Summary and Conclusion

Exercise has acute and chronic modulatory effects on the neural regulation of cardiovascular function. For example, acute exercise causes immediate increases in AP, HR, and SNA that are maintained throughout the exercise session. In contrast, PEH is associated with prolonged vasodilation and has been observed after exercise in healthy and hypertensive subjects. Prolonged endurance training is often accompanied by OI. Baroreflex control of SNA activity is critically involved in the modulation of the AP response to exercise. During exercise, the baroreflex curve for SNA shifts right and upward, allowing simultaneous and sustained increases in AP, HR, and SNA. After exercise, the baroreflex curve for SNA is suppressed vertically, resulting in a sustained reduction of SNA such that AP decreases due to peripheral vasodilation. Prolonged endurance training causes blood volume expansion, which modulates baroreflex control of RSNA in an “Flip-Flop/On-Off” manner. We hypothesized that OI in endurance athletes may be attributable to this “Flip-Flop”-type regulation of SNA. We emphasized in this review that the baroreflex curve for HR does not represent whole body baroreflex function. The arterial baroreflex consists of a multi-input, multi-output, and multilevel control system (Halliwill et al., 1996b; Potts, 2001; Raven et al., 2006; Sagawa, 2011; Matsukawa, 2012; Iwase et al., 2014; Fisher et al., 2015; Dampney, 2017). Beyond the apparent AP changes in response to exercise, modulation of the entire baroreflex curve for SNA differs over various time spans and regions of the body.

## Author Contributions

KM and MY contributed to drafting, revising, and approving the final version of the manuscript.

## Conflict of Interest Statement

The authors declare that the research was conducted in the absence of any commercial or financial relationships that could be construed as a potential conflict of interest.
